# First Episode of Psychosis in a Middle-Aged Patient with a 14-Year History of Conversion Disorder

**DOI:** 10.1155/2014/804930

**Published:** 2014-12-18

**Authors:** Vaios Peritogiannis, Thiresia Manthopoulou, Venetsanos Mavreas

**Affiliations:** ^1^Mobile Mental Health Unit of the Prefectures of Ioannina and Thesprotia, Society for the Promotion of Mental Health in Epirus, 78 Thoma Paschidi, 45445 Ioannina, Greece; ^2^Department of Psychiatry, University of Ioannina School of Medicine, Stavros Niarchos Avenue, 45500 Ioannina, Greece

## Abstract

We present a case of a middle-aged male patient with a long history of conversion disorder and histrionic personality, who presented with newly onset psychotic symptoms while being engaged to treatment with a community mental health team in a primary care setting. The symptoms could not be attributed to an organic cause. After a short course of olanzapine treatment which caused adverse effects, the symptomatology responded well to low dose amisulpride. Conversion symptoms were stable throughout the psychotic episode. This case illustrates the complex interplay between disorders classified in different categories (somatoform versus psychotic disorders).

## 1. Introduction

Cooccurrence of different mental disorders in a patient is challenging for clinical practice. It increases morbidity and may cause disability and makes management more complex than in a single disorder. Comorbidity is common in psychiatric patients; for instance, a study in a primary care setting in Denmark showed that a large proportion up to one-third of patients suffered from two or more mental disorders [1].

Conversion disorder is commonly encountered in neurologic wards but may also involve patients attending a primary care setting. The term refers to a number of symptoms, both motor (such as paralysis, dysphasia, ataxia, tremor, aphonia, and seizures) and sensory symptoms, (diplopia, blindness, deafness, and numbness) which are medically unexplained and presumably have psychogenic origin [2]. Other rare and controversial forms of conversion disorder have been also reported [3, 4]. Such a condition can be frustrating for patients and caregivers and is particularly challenging for clinicians. Course is often chronic, prognosis is generally poor, and disability is common [5]. Conversion disorder has attracted much attention in the past but has received scant recent attention in the psychiatric literature compared with other disorders [2].

On the other hand at recent years there is a growing literature regarding presentation and management of first episode of psychosis. It is now generally believed that the earlier and more comprehensive the intervention, the better the outcome [6–8]. Here we present a case of a middle-aged male patient who developed frankly psychotic symptoms long after a formal diagnosis of conversion disorder with persisted motor symptoms. The patient had been engaged in treatment delivered by the Mobile Mental Health Unit of the Prefectures of Ioannina and Thesprotia (MMHU I-T) and attended regular follow-up appointments in a primary care setting in a rural area of north-west Greece [9, 10].

## 2. Case Presentation

**Figure 1:**
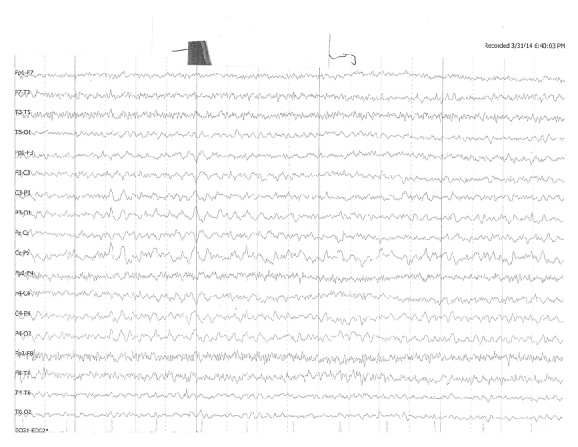
The patient's electroencephalogram had no abnormal findings.

**Figure 2:**
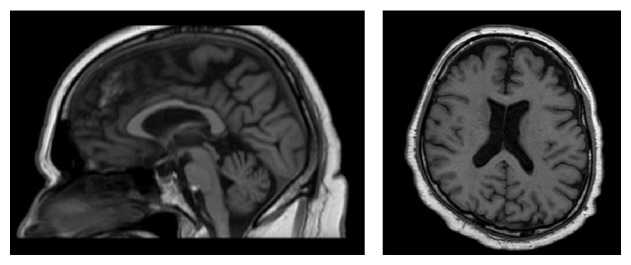
The MRI scan of the brain revealed significant cerebral atrophy.

Mr. A was first examined by the MMHU I-T in 2007, at the age of 50 years. The patient had an established 8-year history of conversion disorder, with onset after a Guillain-Barré syndrome, for which he had been treated in the neurologic ward. His neurologic symptoms had been remitted completely, and repeated electromyogram examinations were normal afterwards. However, he displayed a permanent, though fluctuating leg weakness and although he could walk or even dance in some instances, he mostly used a wheelchair for his transfers. Importantly, the patient received a disability pension. He had been married for 23 years and his wife was a dominant person who had undertaken all the responsibilities. The patient had no history of alcohol or substance misuse. During follow-up he occasionally presented with other conversion symptoms (aphonia) and dissociative symptoms as well (amnesia). Such symptoms usually developed after a stressful life event or even in response to an argument with his wife or other family members. Moreover, the patient was emotionally unstable and histrionic. He often referred to suicidal thoughts but had never attempted suicide. He was receiving antidepressant medication and supportive psychotherapy. After several years of follow-up, with conversion symptoms being stable over time 14 years after the original diagnosis (October 2013) we were informed by his wife that the patient was complaining of hearing voices, telling him that he was the heir of an uninhabited house in the other side of his village. In two occasions he left his home after midnight and went to this house, obeying the voices' commands. He often made a conversation with the voices and believed that these people were watching and conducting him via the internet. Notably, there was no internet connection or a computer in his home. He had also ideas of reference and he believed that these people sent him messages via the television. Although his wife was aware of these symptoms, she informed us about them 4 months after onset. Till then she thought of the patients' claims as part of his usual “nonsense.” The patient was referred for laboratory examinations, including full blood count, neuroimaging of the brain (magnetic resonance imaging, MRI), and electroencephalogram (Figure 1). These examinations revealed a slight vitamin B_12_ deficiency (132 pg/mL, laboratory reference range 145–914 pg/mL) and cerebral atrophy, incompatible with his age (Figure 2). The patient was also assessed with Mini Mental State Examination (MMSE) and scored 30/30. He was prescribed olanzapine titrated up to 15 mg/day, but this compound caused sedation, weight gain, and glucose dysregulation. Subsequently medication was switched to amisulpride 200 mg/day which eliminated psychotic symptoms without adverse effects. He was also prescribed by physicians an oral vitamin B-complex compound for the replacement of B_12_. After several weeks of treatment weight was restored and blood glucose levels were normalized. The patient remains in follow-up and eight months after amisulpride initiation he is free of psychotic symptoms. Conversion symptoms still predominate in clinical picture.

## 3. Discussion

This patient was initially presented as a typical case of conversion disorder; functional neurologic symptoms and secondary gain (i.e., disability pension and no responsibility resumption) were prominent in this case. The long-term course of patient's disorder was in line with literature reports, and he experienced high levels of disability. Prognosis of chronic conversion symptoms is generally poor, and such symptoms often persist and are frequently associated with loss of employment and disability and medical retirement at relatively young age [11–15]. Despite early views of high rates of misdiagnosis of conversion symptoms current evidence suggests that subsequent rediagnosis of neurological disease is uncommon [16]. It is important, however, to search for other psychiatric comorbidities, which is the rule rather than the exception in such patients [17].

Although the diagnosis of conversion disorder has been established at the time of first examination of the patient by our unit, further possibilities as differential diagnosis are worth mentioning. The possibility of factitious disorder (i.e., the intentional production of symptoms with the motivation to obtain medical care and attention) or malingering (which is the intentional production of symptoms to obtain sickness benefits) [14] should be considered. The patient was regularly attending follow-up appointments and was treated as chronic ill, and there was a clear secondary gain for him in the form of disability payments and reduced domestic responsibilities. These conditions may overlap and are likely to be difficult to differentiate in a clinical setting [14]. It has been suggested that while the hallmark of conversion disorder may be inconsistency in physical signs there should not be increased levels of inconsistency in the history relative to other conditions; and when there are major inconsistencies within the history between the patient and an informant or the medical records or between different consultations over time, it could be a sign of feigning [18]. This was not the case of our patient, in favor of the established diagnosis of conversion disorder.

What makes this case interesting and complex is the delayed onset of a first episode of psychosis many years after the original diagnosis of conversion disorder. The patients' symptoms cannot be attributed to B_12_ deficiency. Such deficiency has been reported to induce mental symptoms but psychosis is unusual [19], and in this case deficiency was rather slight (132 pg/mL). Another laboratory finding, revealed by the MRI scan of the brain, was the cerebral atrophy which was incompatible with patient's age. Such changes in brain volume are not uncommon in psychotic patients [20] but are nonspecific and not suggestive of a psychotic disorder. Patients with motor symptoms developed on the context of conversion disorder may undergo neuroimaging of the brain, as part of the diagnostic workup for the exclusion of any lesions. Those patients are unlikely to have gross structural brain changes, but interestingly, in a small study of twelve women with motor conversion disorder, Atmaca et al. [21] found that patients had significantly smaller mean volumes of the left and right basal ganglia and smaller right thalamus, with a trend toward smaller left thalamus compared to healthy controls. Recently, in an MRI study of 15 patients suffering from motor conversion disorder Aybek et al. [22] reported significant cortical thickness increases in the bilateral premotor cortex of hemiparetic patients relative to controls and a trend toward increased grey matter volume in the same region. According to the authors these changes may either represent premorbid vulnerability or more likely a plasticity phenomenon related to the disease. The same investigators found significantly smaller left thalamic volumes in a group of 14 patients compared with controls [23]. On the other hand impairment of cortical and subcortical functioning has been observed with the use of functional neuroimaging [24, 25].

The onset of psychotic disorders is usually in late adolescence and early adult life; however late-onset psychosis may also occur and cause attention from the patient's environment. In this case, the patient's wife thought that patient's complaints of the psychotic symptoms were just nonsense. The patient had been long emotionally unstable, occasionally displayed suicidal claims, and was not taken seriously by his wife. Notably, it has been suggested that when chronic conversion symptoms improve patients may undergo psychiatric decompensation, revealing depression or even previously hidden psychosis [26]. This was not the case of our patient, in whom psychotic symptoms developed without prior improvement of his established conversion symptomatology. On the other hand, in earlier case series there are regular references to diagnoses of schizophrenia, such as the two cases in Slater's influential 1965 series [27]. Perhaps such cases are unusual today because conversion disorder may be rarely seen by psychiatrists. However, in a recent study of 54 patients with conversion disorder who have undergone investigation for psychiatric comorbidity, there was no case of psychotic disorder [28].

After a course of olanzapine treatment resulting in several well-known side effects, the patient was switched to amisulpride. The therapeutic dose was rather low, up to 200 mg/day. The recommended effective dose range for positive symptoms is 400–800 mg/day; lower doses of 100–300 mg/day are used for the treatment of primary negative symptoms. However, there is evidence that, across several age groups, amisulpride may have antipsychotic effects in a broad dosage range, including low doses [29]. It is unknown whether the patients' cerebral atrophy is associated with symptoms' response at the relatively low 200 mg/day dose. Interestingly, there is a reported case of a woman with conversion disorder in whom the administration of amisulpride 200 mg/day resulted in substantial and durable symptom improvement [30]. This was not the case of our patient; however, conversion symptoms remained stable after amisulpride initiation. Given the low treatment dose and the patients' late age it might be argued that psychotic symptoms were developed in the context of a personality disorder. The patient was emotionally unstable and histrionic, and it is known that borderline patients may occasionally display transient, stress-related paranoid ideation. However the patient did not fulfill the DSM-5 criteria for borderline personality disorder, and his psychotic symptoms were not stress-related and were rather schizophreniform. Another diagnostic possibility which would be in line with the MRI finding of cerebral atrophy is that of the prodromal stage of dementia. However, this is not supported by the patient's performance on the MMSE which was as high as 30/30, nor by the overall clinical impression or his spouse reports.

The possibility of the newly onset psychotic features to be conversion symptoms may also be considered. The patient occasionally displayed dissociative symptoms, and conversion and dissociative disorders are in the same section in ICD-10. Moreover, patients with complex and chronic dissociative disorders often experience auditory verbal hallucinations which may be indistinguishable from those experienced by patients with psychotic disorders [31, 32]. However, the dissociative symptoms of our patient were not chronic, and in the current episode he also presented ideas of reference, which supports the diagnosis of a psychotic episode. The possibility that the psychotic symptoms were factitious symptoms cannot be ruled out; however these symptoms elicited anxiety and distress, which would not be expected if the patient was feigning.

Regarding psychotic symptoms remission, it could be argued that they did not in fact respond to the medication but may have remitted spontaneously or they may have remitted due to extinction, because they were not reinforced by the patient's wife and did not result in any additional secondary gain, although, as aforementioned, we do not believe this is the case.

This case is interesting because it illustrates the complex interplay among disorders classified in different diagnostic categories (somatoform versus psychotic disorders). However, the way the two syndromes interact in this case is unknown. Pathophysiological mechanisms at the neurotransmitter level have not been studied in conversion disorder so as to formulate a hypothesis of such patients' proneness to psychotic episodes. It seems plausible that the patient was vulnerable in developing both conversion and psychotic symptoms, but the exact nature of such vulnerability is unknown. It could be argued that psychosis and conversion disorder may share common risk factors other than whatever injury this patient may have suffered as a result of his Guillain-Barré history-life stressors, for example, history of childhood abuse. However, the possibility that the two disorders in this patient were just coincidence cannot be ruled out, and the 14-year interval between the onset of conversion symptoms and the development of psychotic symptoms does tend to argue against a simple cause.

This case also suggests that patients with conversion disorders and certain personality traits may not receive appropriate attention from their family when becoming psychotic, probably because caregivers may be frustrated by patients' complains and the chronic course of such disorders. However, this is important because such lack of attention may result in delayed intervention and this may increase patients' distress and is expected to affect adversely outcome and the long-term prognosis of the psychotic disorder.

It has been suggested that physicians may be afraid of misdiagnosing symptoms as functional in cases of conversion disorder [12]. Along the same lines, treating psychiatrists should be cautious and inquire psychotic symptoms in patients with conversion disorder in cases of alterations in clinical presentation. Such patients may be often perceived as manipulative, dependent, or exaggerating their difficulties [2]. This may result in little appreciation of their psychotic experiences and would prolong untreated illness duration. Treatment of newly onset psychotic symptoms may be much more effective than that of conversion symptoms and thus reliefs patients from additional morbidity and distress and their families from further frustration.
